# Cement augmentation for proximal humerus fractures: a meta-analysis of randomized trials and observational studies

**DOI:** 10.1007/s00068-024-02520-z

**Published:** 2024-04-08

**Authors:** Yannic Lecoultre, Frank J. P. Beeres, Björn C. Link, Fabian Pretz, Franz Tillmann, Reto Babst, Bryan J. M. van de Wall

**Affiliations:** 1 Department of Orthopedic and Trauma Surgery, Lucerne Cantonal Hospital, Lucerne, Switzerland; 2 Faculty of Health Sciences and Medicine, University of Lucerne, Lucerne, Switzerland

**Keywords:** Proximal humerus fracture, ORIF, Plating, Cement augmentation

## Abstract

**Introduction:**

It is unclear if elderly patients treated with plate osteosynthesis for proximal humerus fractures benefit from cement augmentation. This meta-analysis aims to compare cement augmentation to no augmentation regarding healing, complications, and functional results.

**Methods:**

PubMed, Embase, and Cochrane Central Register of Controlled Trials were searched for randomized clinical trials and observational studies. Effect estimates were pooled across studies using random effects models. The primary outcome is overall complication rate. Stratified analyses were performed for types of complication (implant-related or systemic). Secondary outcomes include re-interventions, hospital stay, operation time, functional scores, and general quality of life.

**Results:**

Five observational studies and one randomized controlled trial with a total of 541 patients were included. The overall complication rate was significantly lower in the augmented group (15.6% versus 25.4%, OR 0.54 (95%CI 0.33–0.87)). This was caused by a reduction of implant-related complications (10.4% vs. 19.9%, OR 0.49 (95%CI 0.28, 0.88)). No difference in humeral head necrosis was found. Data on re-intervention, hospital stay, and operation time was limited but did not show significant differences. No impact on functional scores and general quality of life was detected.

**Conclusion:**

This meta-analysis shows that cement augmentation may reduce overall complications, mainly by preventing implant-related complications. No difference was detected regarding need for re-intervention, functional scores, general quality of life, and hospital stay. This is the first meta-analysis on this topic. It remains to be seen whether conclusions will hold when more and better-quality data becomes available.

**Supplementary Information:**

The online version contains supplementary material available at 10.1007/s00068-024-02520-z.

## Introduction

Proximal humerus fractures are the third most common fractures in the elderly, with an incidence of 82 per 100,000 person-years [1]. They are often caused by low-energy trauma in combination with osteoporotic bone structure. The optimal treatment of proximal humerus fractures in elderly patients remains a highly controversial topic. One of the factors complicating treatment decision is poor bone quality itself. There are often concerns whether sufficient purchase and anchorage of screws can be obtained when surgeons opt for osteosynthesis. Loss of reduction and screw cut-out have been described in up to 20% in previous series [2, 3].

Cement augmentation has emerged as a promising technique to reduce these risks. Cannulated/fenestrated screws are used in conjunction with plate osteosynthesis. Cement is injected over these screws and exits at the screw tips to provide superior fixation.

Biomechanical studies have shown that augmentation considerably improves construct stability [4, 5]. The largest point of concern for cement augmentation in proximal humerus fractures is the potential risk of causing humeral head necrosis. Cement can reach temperatures more than 80 °C during ex vivo curing. This leads to the assumption that high temperatures also occur after injection into the bone, with potential corresponding destruction of the surrounding vascularization. Although studies on human bone have shown that the temperature development in vivo usually remains below 45 °C, there is still a certain degree of doubt [6].

There are multiple individual studies on the topic showing mixed results. A formal meta-analysis has not been published yet. The goal of this study is to perform a meta-analysis of all available evidence comparing cement augmentation in plate osteosynthesis of proximal humerus fractures to the same procedure without augmentation. The primary outcome of interest is the overall complication rate. Secondary outcomes include re-interventions, hospital stay, operation duration, range of motion, functional scores, and general quality of life measured at 12 months after the operation.

## Methods

This meta-analysis was performed according to the Preferred Reporting Items for Systematic Reviews and Meta-Analysis (PRISMA) (S2 File) and the Meta-Analysis of Observational Studies in Epidemiology (MOOSE) Guidelines [7, 8]. A standardized approach was used, which is applied in all meta-analyses of our research group [4, 9, 10]. Ethical approval was not required.

The study was recorded at the International Prospective Register of Systematic Reviews (PROSPERO), Nr. CRD42023471427 prior to data collection.

### Search strategy and selection criteria

A comprehensive search in electronic databases (PubMed, Embase, and CENTRAL) for studies on cement augmentation for proximal humerus fractures was performed. Table S1 in the S1 File describes the full search syntax. The search was performed on December 1, 2023. All randomized controlled trials and observational studies that compared cement augmentation with no augmentation in plate fixation of proximal humerus fractures were included in this review. Other inclusion criteria were reporting on the outcomes of interest and availability of full text.

Exclusion criteria were cadaveric studies, studies on pathologic fractures, case reports, and studies with languages other than English, Dutch, French, German, Spanish, or Italian.

Two reviewers assessed the search and inclusion of studies independently (YL, BW). Disagreement was solved by consensus with a third reviewer (FB).

### Data extraction

Study and patient characteristics were collected in a predefined data extraction sheet and included first author, publication year, country/region in which the study was performed, study design, study population size, operating position/approach, and type of cement used. Furthermore, fracture type according to the Neer classification, gender, and the patient’s history of smoking or diabetes were extracted [11].

### Quality assessment

The same two reviewers (YL, BW) assessed the methodological quality of included studies, independently using the MINORS criteria [12]. Disagreement was resolved by consensus. Details are described in Table S2 in the S1 File.

### Primary outcome

The primary outcome was overall complication rate. Additionally, complications were subdivided in fracture/implant-related and systemic. Fracture/implant-related complications included screw perforation/cut-out, total or partial humeral head necrosis, non-union, loss of reduction, implant bending or breakage, and deep wound infection. Non-union was defined as the absence of callus formation or fading of fracture lines on X-ray after 6 months. Loss of reduction was ≥ 15° increase of varus malposition and a relative change of ≥ 5 mm of the greater or lesser tuberosity [13].

Systemic complications included thromboembolic complications, pneumonia, and renal insufficiency. Also, a separate analysis specifically addressing the risk of humeral head necrosis and screw perforation was performed.

### Secondary outcomes

Secondary outcomes included re-interventions, hospital stay, operation time, range of motion, functional scores, and general quality of life measured at 12 months after the operation. Re-interventions included all re-operations performed on the affected fracture site during follow-up. Functional scores included Constant score and DASH score [14, 15]. General quality of life was measured using the EQ5D score [16].

### Statistical analysis

Continuous variables were presented as means with standard deviation (SD) or range. If required, information was converted to mean and SD using the methods described in the Cochrane Handbook for Systematic Reviews of Interventions. Dichotomous variables were presented as counts and percentages.

Formal meta-analysis was only performed in case three or more studies reported on the outcome of interest. Effects of treatment options on continuous outcomes were pooled using the (random effects) inverse variance weighting method. They were presented as mean difference (MD) with corresponding 95% confidence interval (95%CI). Binary outcomes were analyzed using the (random effects) Mantel–Haenszel method. They were presented as odds ratio (OR) or risk difference (RD), with a 95%CI.

Heterogeneity between studies was quantified by the *I*^2^ statistic. The threshold for significance was set at a *p*-value of 0.05. Review Manager (RevMan, version 5.4.1) was used for statistical analysis.

## Results

### Literature search

A total of 651 references were evaluated. A detailed description of the search and screening is shown in Table S1 in the S1 File. Five observational studies and one randomized controlled trial fulfilled the criteria [13, 17–21].

### Baseline characteristics

The six studies included a total number of 541 patients, of which 249 received cement augmentation following fixation. Baseline characteristics including age, gender, Neer classification, operative approach, and type of cement are described in Table 1. They were equally distributed among treatment groups. All studies used a deltopectoral approach. Polyaxial locking-compression plates were used in all studies.

**Table 1:** Baseline characteristics. *SD* standard deviation, *nr* not reported, *OS* observational study, *RCT* randomized controlled trial, *CA* cement-augmented, *N-CA* non-cement-augmented

First author	Year	Country/region	Design	Approach	Cement type	Follow-up (months)	Inclusion	Number of cases		Age mean (SD)		Gender (female, %)		Neer classification (2-/3-/4- part), %					
								CA	N-CA	CA	N-CA	CA	N-CA	CA2	CA3	CA4	N-CA2	N-CA3	N-CA4
Egol	2012	USA	OS	Deltopect	CaPhos	12	63	27	36	61	58	nr	nr	18	67	15	44	56	0
Katthagen	2018	Germany	OS	Deltopect	PMMA	12	48	24	24	74.2 (10.1)	73.9 (9.4)	91.7	91.7	nr	nr	nr	nr	nr	nr
Hengg	2019	Europe	RCT	Deltopect	PMMA	12	67	33	34	77.5 (7.4)	76.1 (6.2)	78.8	85.3	3	48.5	48.5	14.6	50	35.3
Siebenbuerger	2019	Germany	OS	Deltopect	PMMA	24	94	39	55	78.2 (10.2)	76.6 (11.1)	82.2	78.1	41	38.5	20.5	36.4	40	23.6
Foruria	2021	Spain	OS	Mod. Deltopect	PMMA	12	168	78	90	76 (8)	76 (6)	83	79	15.4	33.3	41	14.4	53.3	14.4
Hakimi	2021	Germany	OS	Deltopect	PMMA	9	101	48	53	76.3 (8.6)	72.8 (7.1)	83	83.3	12	88	0	47	53	0

### Quality assessment

The mean quality of all studies was 19 points (range 16–22) using the MINORS criteria. Details can be found in Table S3 in the S1 File.

### Primary outcome

**Fig. 1:**
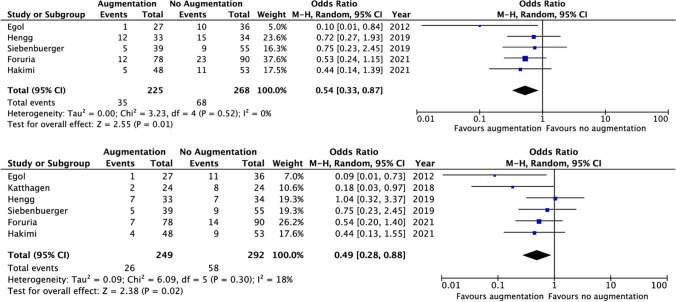
Forest plots of overall and implant-related complications

Overall complications were reported in five studies: one randomized clinical trial and four observational studies. The overall complication rate was significantly lower in the augmented group (15.6% versus 25.4%, RD 11%, 95%CI 4–18%) with an OR of 0.54 (95%CI 0.33–0.87, *I*^2^ 0%) (Fig. 1).

**Fig. 2:**
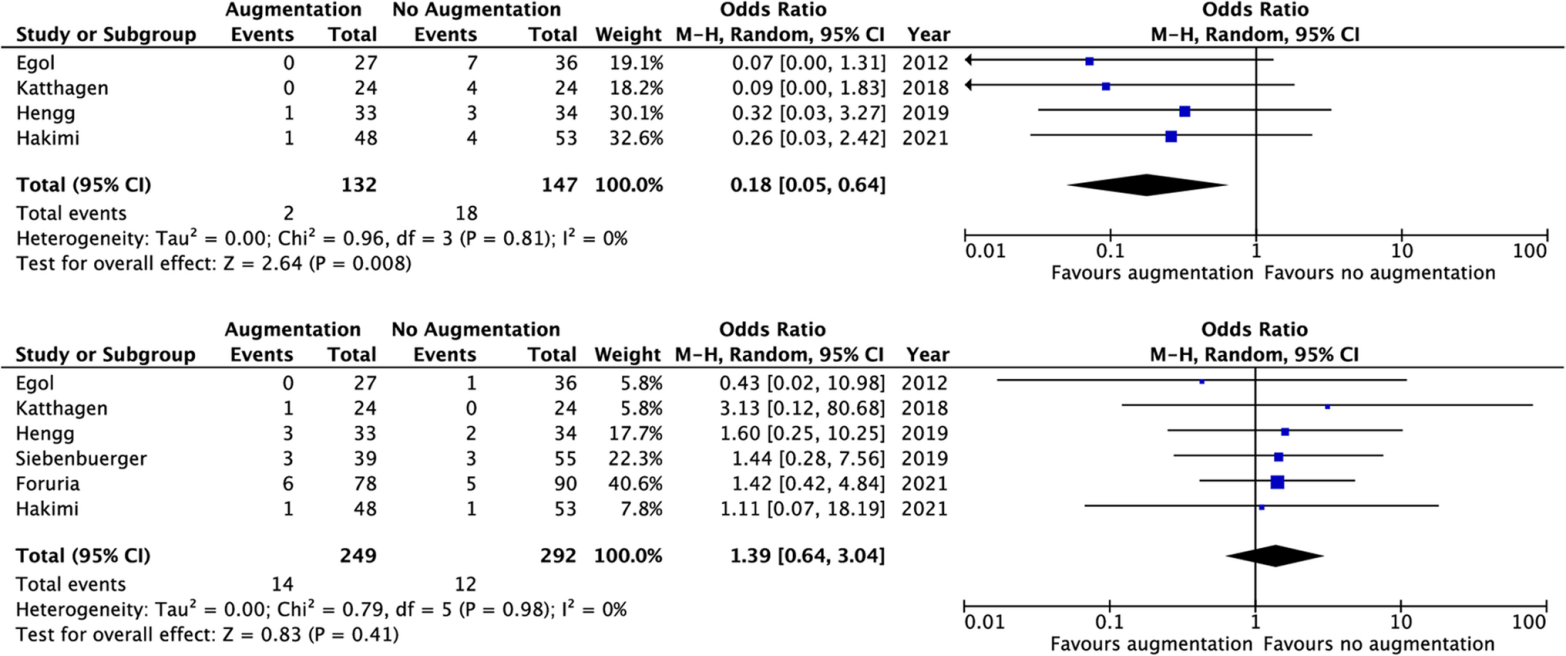
Forest plots of screw penetration and humeral head necrosis

The difference in overall complications between treatment groups was predominantly caused by a difference in implant-related complications, which occurred in 10.4% in the augmented versus 19.9% in the non-cemented group with an OR of 0.49 (95%CI 0.28, 0.88, *I*^2^ 18%). All implant-related complications are described in Table 2. The most common complication was secondary screw protrusion, which occurred more frequently in the non-cemented group (1.5% vs. 12.2%, OR 0.18, 95%CI 0.05–0.64, *I*^2^ 0%) (Fig. 2). Loss of fixation was not significantly different between the groups (3.3% in the augmented group vs. 7.7% in the non-augmented group, OR 0.43, 95% CI 0.13, 1.39, *I*^2^ 0%). There was no significant difference in humeral head necrosis (5.6% in the augmented vs. 4.1% in the non-augmented group, OR 1.39, 95%CI 0.64, 3.04, *I*^2^ 0%).

**Table 2:** Implant-related complications. *nr* not reported, *CA* cement-augmented, *N-CA* non-cement-augmented

First author	Year	Number of cases		Screw perforation		Implant failure		Mal/non-union		Humerus head necrosis		Loss of fixation		Deep wound infection	
		CA	N-CA	CA	N-CA	CA	N-CA	CA	N-CA	CA	N-CA	CA	N-CA	CA	N-CA
Egol	2012	27	36	0	7	0	1	0	1	0	1	nr	nr	1	1
Katthagen	2018	24	24	0	4	0	0	1	4	1	0	nr	nr	0	0
Hengg	2019	33	34	1	3	0	0	1	0	3	2	1	2	1	0
Siebenbuerger	2019	39	55	nr	nr	0	0	nr	nr	3	3	2	6	0	0
Foruria	2021	78	90	nr	nr	1	8	0	1	6	5	nr	nr	0	0
Hakimi	2021	48	53	1	4	nr	nr	nr	nr	1	1	1	3	1	1
Total		**249**	**292**	**2**	**18**	**1**	**9**	**2**	**6**	**14**	**12**	**4**	**11**	**3**	**2**
Total (%)				1.5	12.2	0.5	3.8	1.2	3.3	5.6	4.1	3.3	7.7	1.2	0.7

### Secondary outcomes

#### Re-intervention

**Fig. 3:**
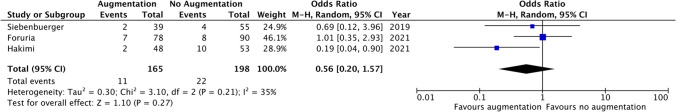
Forest plot of re-intervention

Three studies reported on re-intervention. Re-intervention was performed in 6.7% in the augmented vs. 11.1% in the control group. This difference was not significant (OR 0.56 [0.20, 1.57], *p* 0.21) (Fig. 3).

#### Hospital stay

The duration of hospital stay was reported in one study only. There was no difference between the groups with a mean of 9.1 days (SD 3.1) in the cement-augmented group and 9.2 (SD2.9) in the non-augmented group.

#### Operation time

Two studies reported on operative time. In one study, no difference was found. The mean duration was 69 min (SD 17 min) in the cemented and 68 min (SD 23 min) in the non-cemented group. The other study found a significant difference with a longer operation duration in the cemented group (mean 105, SD 26 min in the cemented group versus 95, SD 26 min in the non-cemented group).

#### Functional scores

**Fig. 4:**
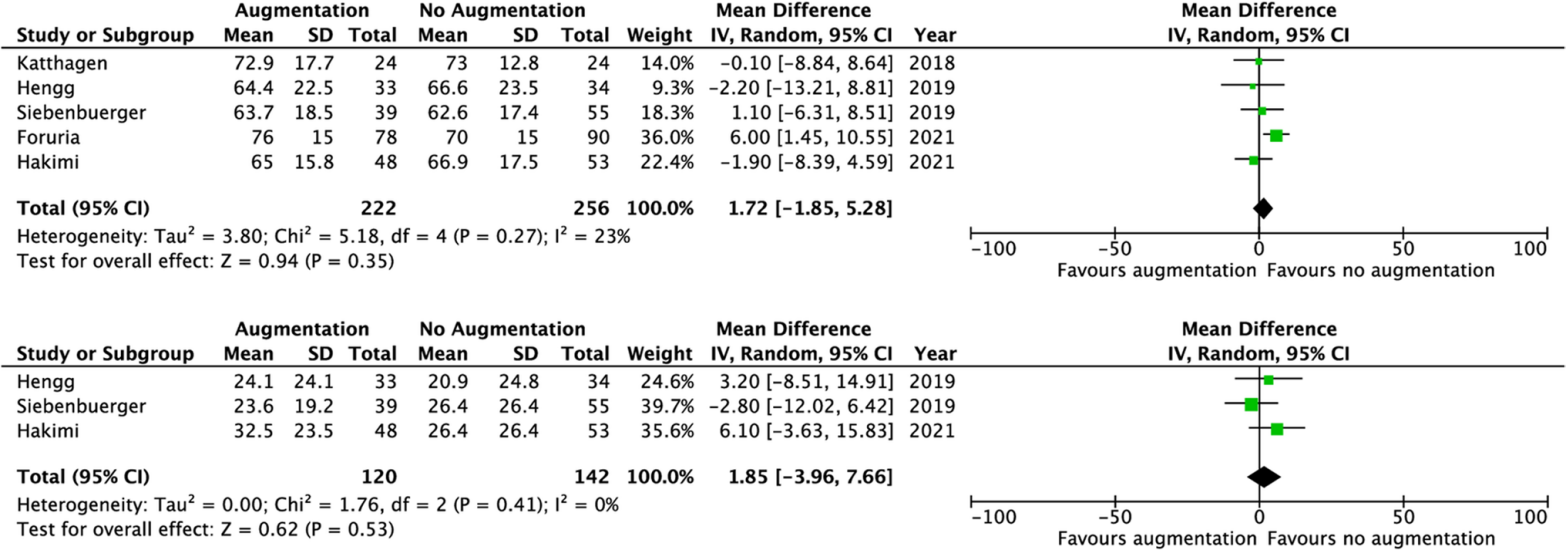
Forest plots of Constant score and DASH score

Five studies reported on the Constant score and three on the DASH score measured 9–12 months after surgery. Both scores showed no significant differences between groups. The Constant score was 69.4 in the cemented versus 67.6 in the non-cemented group (MD 1.72 [− 1.85, 5.28], *p* 0.27, *I*^2^ 23%). The DASH score was 27.3 in the cemented versus 25.1 in the non-cemented group (MD 1.85 [− 3.96, 7.66], *p* 0.41, *I*^2^ 0%) (Fig. 4).

#### General quality of life

One study assessed quality of life 12 months postoperatively using the EQ5D questionnaire. No significant differences could be shown (0.85 (95%CI 0.77; 0.93) vs. 0.90 (95%CI 0.82; 0.98)).

## Discussion

This meta-analysis of one randomized clinical trial and five observational studies compared cement augmentation to no augmentation in fixation of proximal humerus fractures in elderly patients. Cement augmentation appears to lead to fewer peri- and postoperative complications (15.6% versus 25.4%). This is mainly caused by a decrease in implant-related complications (10.4% vs. 19.9%) of which secondary screw protrusion is the most common. No obvious negative effects of cement augmentation, such as an increase in humeral head necrosis, could be demonstrated. No difference in need for re-intervention, functional scores, general quality of life, and hospital stay was detected.

### Comparison with literature

To date, this is the first formal meta-analysis on this topic. A systematic review on the use of cement augmentation in proximal humerus fractures including clinical and biomechanical studies was published in 2020 [5]. The authors pointed out the possible benefits of the technique in terms of stability (i.e., citing the reduction in screw penetration from 16.6 to 8% in the study of Katthagen et al. or the loss of fixation in 10.9% (cemented) vs. 5.1% (non-cemented) reported by Siebenbuerger et al.). Due to the limited clinical data, however, no clear recommendation could be made. Additionally, concerns regarding the safety of the procedure, mainly the risk of cement-related humeral head necrosis, were pointed out. With two new large-scale studies published in the past 2 years, almost double the number of patients were available creating the opportunity to investigate the benefit of cement on a meta-analytical level. With this dataset, the possible benefits and the safety of the procedure in terms of cement-related complications could be investigated more thoroughly.

### Interpretation of results

Based on the results of this meta-analysis, one could argue that cement augmentation should be routinely used in elderly patients treated with plate fixation for proximal humerus fractures. However, certain aspects should be considered.

The benefit of cement augmentation appears to lie predominantly in reducing the risk of implant failure, more specifically secondary screw protrusion. Interestingly, one would expect to find a lower re-intervention rate in the cemented group. This, however, is not the case. A logical explanation might lie in the study population itself. Elderly patients with proximal humerus fractures generally have an advanced age and low demands. The threshold to perform a second operation in this fragile patient group is high. These characteristics combined with the fact that implant failures do not necessarily cause severe complaints might reduce the need and desire for revision surgery. On the other hand, the fact that no difference in re-intervention was found might very well be caused by underpowering as only three studies reported on this outcome.

Regarding adverse effects, a major concern in cement augmentation is humeral head necrosis. This meta-analysis showed no significant difference regarding this complication. All studies, however, did point in the same direction indicating that it might occur more often in the cemented group. Nevertheless, the absolute risks in this meta-analysis were low (5.6% in the augmented vs. 4.1% in the non-augmented group). Indeed, much higher risks up to 35% have been described in previous literature [22]. This is however mainly attributable to the fact that their study population contained more four-part fractures, a well-known risk factor for humeral head necrosis, than included in the present meta-analysis. In other words, it is plausible to assume that humeral head necrosis is mostly dictated by fracture morphology rather than whether cement augmentation was used or not.

Regarding the functional results, no difference was found with little heterogeneity. This seems a logical finding as cement mainly has a biomechanical advantage. It reinforces the construct and does not directly improve functional outcomes such as range of motion. Also, functional results were measured at approximately 1 year. It is a well-known fact that measuring functional scores at 1 year follow-up carries the risk of measuring coping instead of true function. The best possible time to measure functional scores is 6 months. This data, regrettably, was not available in this meta-analysis.

It should be questioned whether the additional costs of cement augmentation including the costs for fenestrated/cannulated screws are justified. Indeed, it reduces the risk of implant failure; however, it remains uncertain whether this has any clinical consequences. Material costs of cement augmentation are approximately 500$ [23]. Costs related to a potential increased operation duration seem negligible based on the results of this meta-analysis.

To our opinion, cement augmentation is justified, but whether to use it should be determined on a case-by-case basis. It should be underlined that cement augmentation cannot be used as a salvage procedure in poorly reduced fractures. Good patient selection and anatomical reduction of the fracture remain the most decisive factors in preventing complications. To our opinion, patients that will benefit the most from it are those with a fracture pattern with acceptable risks for humeral head necrosis where anatomic reduction is achieved but the osteosynthesis requires additional anchorage due to poor bone quality or little bony substance surrounding the screw tips.

### Limitations

In this meta-analysis, observational studies were mainly included. Only one randomized clinical trial was available. Although previous meta-analyses have shown that pooled analysis of observational studies demonstrates similar risk estimates as the ones of randomized clinical trials in orthopedic trauma research, this assumption could not be internally validated in the present meta-analysis. Furthermore, it should be noted that heterogeneity in some of the analyses was quite high, i.e., for re-intervention and operative time, making these results less reliable.

Furthermore, the quality of the studies included varied considerably. Many also lacked clear definitions on study outcomes such as humeral head necrosis and wound infection.

## Conclusion

This meta-analysis shows that cement augmentation in plate fixation of proximal humerus fractures in elderly patients may have a beneficial effect on overall peri- and postoperative complications, mainly by reducing the risk implant-related complications such as secondary screw protrusion. No increased risk of humeral head necrosis was found when compared to non-augmented patients. Also, no difference was detected regarding the need for re-intervention, functional scores, general quality of life, and hospital stay. This is the first meta-analysis on this topic. It remains to be seen whether conclusions will hold when more and better-quality data becomes available in the near future.

## Supplementary Information

Below is the link to the electronic supplementary material.

Supplementary file1 (DOCX 26 KB)

Supplementary file2 (DOCX 32 KB)

## Data Availability

No datasets were generated or analyzed during the current study.

## Abbreviations

SD: Standard deviation
CI: Confidence interval
OR: Odds ratio
MD: Mean difference
