# Supplementary material for: Cement augmentation for proximal humerus fractures: a meta-analysis of randomized trials and observational studies
Source: Eur J Trauma Emerg Surg. 2024 Apr 8;50(5):2053–60. doi: 10.1007/s00068-024-02520-z (PMC11599401; doi:10.1007/s00068-024-02520-z)
Supplement: Supplementary file 1 — Supplementary file1 (DOCX 26 KB) [file 68_2024_2520_MOESM1_ESM.docx]

S1 File

Cement augmentation for proximal humerus fractures: A meta-analysis of randomized trials and observational studies

Yannic Lecoultre^1,2*^, Frank JP Beeres^1,2^, Björn C Link^1^, Fabian Pretz^1,2^, Franz Tillmann^1^, Reto Babst^1,2^, Bryan JM van de Wall^1,2.^

1 Department of Orthopedic and Trauma Surgery, Lucerne Cantonal Hospital, Lucerne, Switzerland

2 Faculty of Health Sciences and Medicine, University of Lucerne, Lucerne, Switzerland

* Corresponding Author

Yannic Lecoultre

y@lecoultre.cc

##

## S1 Table: Search Strategy / Syntax

| **Pubmed/MEDLINE (n=412)**  **Embase (n=175)**  **CENTRAL (n=64)**  **Total (n=651)** |  |
| --- | --- |
| **Title and abstract screening (n=15)** | Excluded by title / abstract: n=636 |
| **Full text articles assessed for eligibility (n=7)** | Non-comparative studies: n=1 |
| **Studies included (n=6)** |  |

| **Pubmed/MEDLINE** | ((cement*[Title/Abstract]) OR (augment*[Title/Abstract])) AND (proximal humer*[Title/Abstract]) |
| --- | --- |
| **Embase** | ('proximal humerus fracture'/exp OR 'proximal humerus fracture') AND cement* |
| **CENTRAL** | #1 ("proximal humeral fracture"):ti,ab,kw (Word variations have been searched) 209  #2 (*cement):ti,ab,kw OR ("augmentation"):ti,ab,kw (Word variations have been searched) 110544  #3 #1AND#2 |

## S2 Table: Quality assessment criteria

| **Criteria** | **Reported and adequate (2)** | **Reported but inadequate (1)** | **Not reported (0)** |
| --- | --- | --- | --- |
|  |  |  |  |
| Clearly stated aim | Aim including outcomes reported | Aim reported without outcomes | Not reported |
| Inclusion consecutive patients | Inclusion of consecutive patients | Clear description of inclusion criteria | Not reported |
| Prospective collection data | Prospective | retrospective | Not applicable |
| Appropriate endpoints | Appropriate endpoints to aim study | Endpoints not appropriate to aim study | Not reported |
| Unbiased assessment | Blinded evaluation of outcomes | Reason not blinding stated | Not reported |
| Appropriate follow-up | Minimum follow-up 12 months | Follow-up < 12 months | Not reported |
| Loss to follow-up < 5% | Less than 5 % | Reported, more than 5% | Not reported |
| Prospective calculation study size | Prospective power-analysis performed | Power analysis performed but inadequate | Not applicable |
| Adequate control group | Operative versus nonoperative treatment | Not applicable | Not applicable |
| Contemporary groups | Study/control group managed during same period | Study/control not managed during same period | Not reported |
| Baseline equivalence groups | Baseline characteristics described and comparable | Baseline characteristics not comparable | Not reported |
| Adequate statistical analyses | Statistical analysis described including type of analyses | Inadequate description statistical analysis | Not reported |

## S3 Table: Quality assessment

|  | Egol | Katthagen | Hengg | Siebenbuerger | Foruria | Hakimi |
| --- | --- | --- | --- | --- | --- | --- |
| **Clearly stated aim** | 2 | 2 | 2 | 2 | 2 | 2 |
| **Inclusion of consequetive patients** | 2 | 1 | 2 | 2 | 2 | 2 |
| **Prospective data collection** | 1 | 1 | 2 | 1 | 1 | 1 |
| **Appropriate endpoints** | 2 | 2 | 2 | 2 | 2 | 2 |
| **Unbiased assessment endpoints** | 0 | 0 | 2 | 0 | 0 | 2 |
| **Appropriate follow-up** | 2 | 2 | 2 | 2 | 2 | 2 |
| **Loss-to-follow-up <5%** | 0 | 2 | 1 | 1 | 1 | 0 |
| **Prospective calculation study size** | 2 | 0 | 1 | 2 | 0 | 1 |
| **Adequate control group** | 2 | 2 | 2 | 2 | 2 | 2 |
| **Contemporary groups** | 2 | 0 | 2 | 2 | 2 | 2 |
| **Baseline equivalence of groups** | 2 | 2 | 2 | 2 | 2 | 2 |
| **Adequate statistical analysis** | 2 | 2 | 2 | 2 | 2 | 2 |
| **Total:** | 19 | 16 | 22 | 20 | 18 | 20 |
